# Identifying Hemodialysis Patients With the Highest Risk of *Staphylococcus aureus* Endogenous Infection Through a Simple Nasal Sampling Algorithm

**DOI:** 10.1097/MD.0000000000003231

**Published:** 2016-04-08

**Authors:** Paul O. Verhoeven, Julie Gagnaire, Cyrille H. Haddar, Florence Grattard, Damien Thibaudin, Aida Afiani, Céline Cazorla, Anne Carricajo, Christophe Mariat, Eric Alamartine, Frédéric Lucht, Olivier Garraud, Bruno Pozzetto, Elisabeth Botelho-Nevers, Philippe Berthelot

**Affiliations:** From the GIMAP EA 3064 (Groupe Immunité des Muqueuses et Agents Pathogènes) (POV, JG, CHH, FG, AC, CM, EA, FL, OG, BP, EB-N, PB), University of Lyon, 42023 Saint-Etienne; Laboratory of Infectious Agents and Hygiene (POV, CHH, FG, AC, BP, PB); Infectious Diseases Department (JG, CC, FL, EB-N, PB); Nephrology-Dialysis-Transplantation Department (DT, CM, EA), University Hospital of Saint-Etienne, 42055 Saint-Etienne Cedex 02; and ARTIC42 Center (AA), 42270, Saint-Priest en Jarez, France.

## Abstract

In contrast to *Staphylococcus aureus* intermittent nasal carriers, persistent ones have the highest risk of infection. This study reports the usefulness of a simple nasal sampling algorithm to identify the *S. aureus* nasal carriage state of hemodialysis patients (HPs) and their subsequent risk of infection.

From a cohort of 85 HPs, 76 were screened for *S. aureus* nasal carriage once a week during a 10-week period. The *S. aureus* nasal load was quantified by using either culture on chromogenic medium or fully automated real-time polymerase chain reaction assay. Molecular typing was used to compare strains from carriage and infection.

The algorithm based on quantitative cultures was able to determine the status of *S. aureus* nasal carriage with a sensitivity of 95.8%, a specificity of 94.2%, a positive predictive value of 88.5%, and a negative predictive value of 98.0%. Of note, the determination of the *S. aureus* carriage state was obtained on the first nasal sample for all the 76 HPs, but 1 (98.7%). The algorithm based on quantitative polymerase chain reaction assay directly from the specimen yielded similar performances. During the 1-year follow-up after the last sampling episode, HPs classified as persistent nasal carriers with the algorithm were found to have a higher risk of *S. aureus* infection than those classified as nonpersistent carriers (*P* < 0.05), especially for infections of endogenous origin (*P* < 0.001).

This simple algorithm is reliable for determining the *S. aureus* nasal carriage status in clinical practice and could contribute to characterize at an early stage of take-up patients with the highest risk of *S. aureus* infection.

**KEY FINDING**

- This prospective study reports the clinical validation of an algorithm based on 1 or 2 quantitative nasal samples for reliably predicting at an early stage the *S. aureus* nasal carriage state and the subsequent risk of infection in hemodialysis patients.

## INTRODUCTION

The nasal carriage of *Staphylococcus aureus* is a major risk factor of *S. aureus* infection, notably in hemodialysis patients (HPs).^1^ A recent meta-analysis confirmed that decolonization strategies using mupirocin were able to reduce the rate of *S. aureus* infection in this category of patients,^2^ although the risk of emergence of antimicrobial resistance persists.^3,4^ Approximately one-quarter of the general population is colonized by *S. aureus* in the anterior part of the nostril (*vestibulum nasi*).^5^ Three main categories of nasal carriers have been historically identified: persistent carriers (20% [12%–30%]), intermittent carriers (30% [16%–70%]), and noncarriers (50% [16%–69%]).^1,6^ By contrast to intermittent carriers and noncarriers, persistent nasal carriers have a higher risk of *S. aureus* infection, especially those in continuous peritoneal dialysis^7^ and in orthopedic surgery.^8^

Persistent carriers are characterized by a higher nasal bacterial load,^9,10^ a longer duration of carriage,^11^ a lower rate of exchange of *S. aureus* strains,^12,13^ and a particular affinity for the carried strain.^11^ However, there is no consensual definition of this persistent carriage state. In previous studies aimed to define the *S. aureus* carriage state, 5 to 12 nasal sampling episodes were realized for a period ranging from 5 weeks to 8 years.^7,10,13,14^ The index of carriage, corresponding to the number of samples positive for *S. aureus* divided by the total number of samples, has been proposed to standardize the definition of the carriage state.^13^ According to these reference tools, it is almost impossible to determine the nasal carriage state in routine practice. Nouwen et al^9^ proposed a “culture rule” based on 2 qualitative cultures from nasal specimens taken 7 days apart, with positive and negative predictive values of 0.79 and 0.99, respectively, for distinguishing persistent and nonpersistent nasal carriers. This approach was used mainly for research purposes^5^ because it remains difficult to obtain systematically 2 nasal samples within 1 week in clinical practice.

Recently, we described an algorithm based on 1 or 2 quantitative cultures from nasal samples taken within 2 days that was able to distinguish accurately persistent and nonpersistent nasal carriers of *S. aureus*; only 1 nasal sample was needed in more than 9 cases out of 10.^10^ The aim of the present study was to assess prospectively the reliability of this algorithm in clinical practice in a cohort of HPs and to check its ability for identifying patients with the highest risk of *S. aureus* infection.

## METHODS

### Population

The study was proposed to HPs of the University Hospital of Saint-Etienne, France, and of the nearby ambulatory hemodialysis center ARTIC 42, between January 2012 and March 2013. Included subjects were aged 25 years or older and possessed a health insurance. They were not enrolled if at least 1 of the following exclusion criteria was recorded: carrier state of *S. aureus* already determined; chronic soft skin tissue infection due to *S. aureus* or eczema; ongoing or completed antibiotic treatment for less than 15 days; nasal decolonization by mupirocin or skin decolonization by antiseptic bath for at least 5 consecutive days in the previous year; pregnancy; HIV infection; and hemostasis disorder. Secondary exclusion criteria were as follows: 2 first nasal samples taken in more than 7 days; follow-up after inclusion of less than 10 weeks; less than 7 sampling episodes; and declaration of pregnancy after inclusion. For patients treated with antimicrobials during the follow-up, the nasal screening was discontinued and started again at least 15 days after the end of the treatment.

### Nasal Sampling Procedure

Nasal samples were taken by the nursing staff of the ward using nylon flocked swab (eswab, ref 480CE, Copan, Brescia, Italy). Before taking the sample, swabs were wetted using an additional tube containing a sponge impregnated with normal saline solution and manufactured by Copan for the study purposes. Except for a few numbers of samples (less than 5%) that were stored at 4°C for a few hours, the samples were processed immediately after sampling because of the vicinity between the 2 hemodialysis centers and the laboratory.

### Microbiological Methods

A 100-μL volume of nasal specimen was plated onto chromogenic agar (CHROMagar Staph aureus, Becton Dickinson). The identification of presumptive colonies was confirmed by using Microflex LT mass spectrometer (Bruker Daltonics, Bremen, Germany). The *S. aureus* nasal load of the first 2 swabs was determined by both culture onto chromogenic agar and full automated quantitative polymerase chain reaction (qPCR) (Xpert MRSA/SA Nasal, Cepheid) as previously described.^15^

### Definition of the Reference *Staphylococcus aureus* Nasal Carriage State

The HPs were sampled over a 10-week period using at least 7 and at most 12 sampling episodes. *S. aureus*-persistent nasal carriers were defined by an index of carriage greater than or equal to 0.8, and *S. aureus*-intermittent nasal carriers by a positive index of carriage lower than 0.8.^13^

### *Staphylococcus aureus* Nasal Carriage State Defined by the Algorithm

The algorithm used in the study was previously built from healthcare workers.^10^ Subjects were classified as persistent nasal carriers if the *S. aureus* nasal load was greater than 10^3^ colony-forming unit (CFU)/swab on the first swab or greater than 10^2^ CFU/swab on the first 2 nasal swabs. In other cases, they were classified as nonpersistent carriers.

### *Staphylococcus aureus* Infection in HPs

*S. aureus* infection in HPs was prospectively recorded during 1 year after the last sampling by hemodialysis practitioners. The files of the microbiology laboratory were also screened for identifying potential infection due to this bacterium. The definite validation of *S. aureus* infection was established by an infectious disease specialist (EBN). The relationship between the strains isolated from colonization and infection was established by using arbitrarily-primed PCR, spa-typing, and DNA microarray (StaphyType kit, Alere, Jouy-en-Josas, France), as previously described.^16–18^

### Statistical Analysis

Descriptive variables, sensitivity, specificity, and predictive values were reported with their 95% confidence interval (CI). Parametric and nonparametric tests were performed using MedCalc software v13.1.2 (Ostend, Belgium). *P* values under 5% were considered to be statistically significant.

### Ethical Statement

This study was approved by the regional ethical committee (“Comité de protection des personnes Sud-Est 1”). All the participants gave their informed written consent before entry into the study.

## RESULTS

### Study Population

**FIGURE 1:**
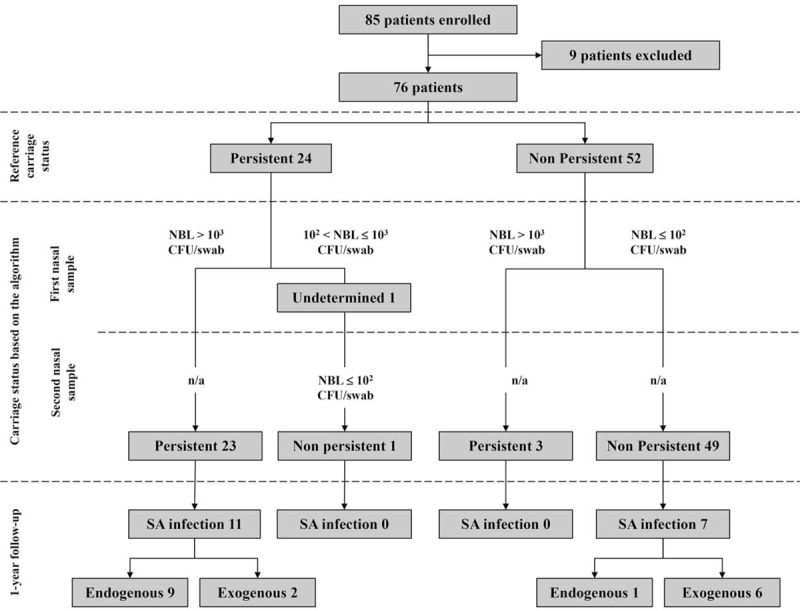
Flow chart of the study. n/a = not applicable because the nasal carriage status was already assigned with the first nasal sample, NBL = nasal bacterial load of *Staphylococcus aureus*, SA = *S. aureus*.

Eighty-five HPs were enrolled into the study between January 2012 and March 2013. Nine of them were excluded secondarily according to the criteria described in the above section. Consequently, 76 HPs were included in the data analysis (mean age 70.0 years, SD 11.0; M/F sex ratio 1.37). Three of them received antimicrobial drugs during the follow-up period; as mentioned above, the nasal screening was stopped during the treatment and was restarted at least 15 days after the cessation of antimicrobial therapy. The flow chart of the study is depicted in the Figure 1.

### Prospective Validation of the Algorithm in Clinical Routine Practice

First, the reference nasal carriage status of each HP was determined by using a mean of 11.4 samples per patient (SD 0.7) over a mean period of 10.3 weeks (SD 0.6). The median and the mean delays between the first 2 consecutive samples were 2.0 and 2.2 (SD 0.4) days, respectively. A total of 44 *S. aureus* nasal carriers (57.9%) were identified during the period of follow-up, including 24 persistent carriers (31.6%) and 20 intermittent carriers (26.3%) (Figure 1). Methicillin-resistant *S. aureus* was identified in 11 nasal carriers (14.5%), including 9 persistent and 2 intermittent ones. The *S. aureus* nasal load was found higher in patients with persistent nasal carriage (mean of 6.0 log CFU/swab) than in patients with intermittent nasal carriage (mean of 1.1 log CFU/swab) (*P* < 0.0001 by Welch test). The algorithm based on quantitative culture was applied and found able to classify persistent and nonpersistent carriers with a sensitivity, specificity, positive predictive value, and negative predictive value (95% confidence interval [CI] of 95.8% (95% CI 78.9–99.9), 94.2% (95% CI 84.1–98.8), 88.5% (95% CI 69.9–97.6), and 98.0% (95% CI 89.4–100), respectively. Identical results were obtained with the qPCR assay (Table 1). The determination of the *S. aureus* carriage state was obtained on the first nasal sample for all the 76 HPs, but 1 (98.7%) (Figure 1). As shown in Table 2, 3 nonpersistent carriers were misclassified as persistent carriers by using the algorithm, whereas the opposite pattern was observed in 1 case. It is worthwhile to note that 2 of the 3 carriers classified as persistent with the algorithm exhibited an index of carriage greater than 0.7 (Table 2).

**TABLE 1:**
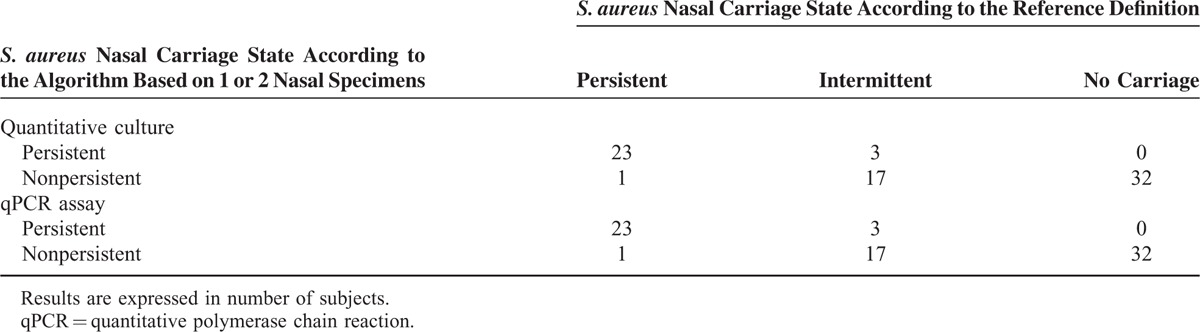
Performances of the Algorithm Based on 1 or 2 Nasal Samples

**TABLE 2:**
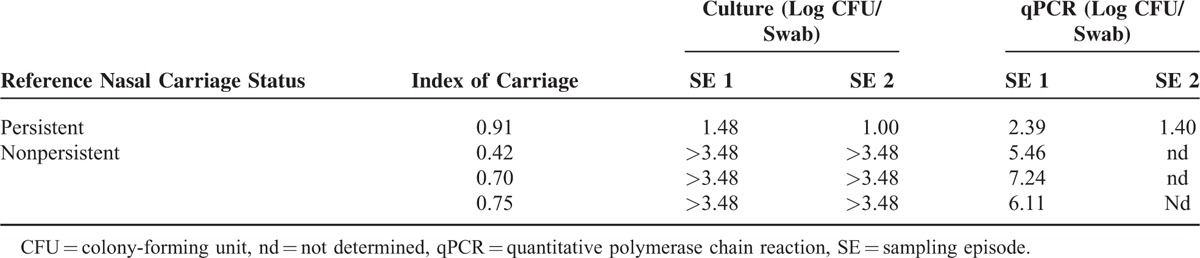
Discrepant Results Between the Reference Definition of Nasal Carriage State and the Algorithm Based on Quantitative Culture or qPCR

Table 3 describes the demographic and clinical characteristics of the group of persistent carriers compared with that of nonpersistent carriers according to the algorithm based on 1 or 2 nasal samples. No statistically significant difference was observed between the 2 groups for any of the items listed in Table 3.

**TABLE 3:**
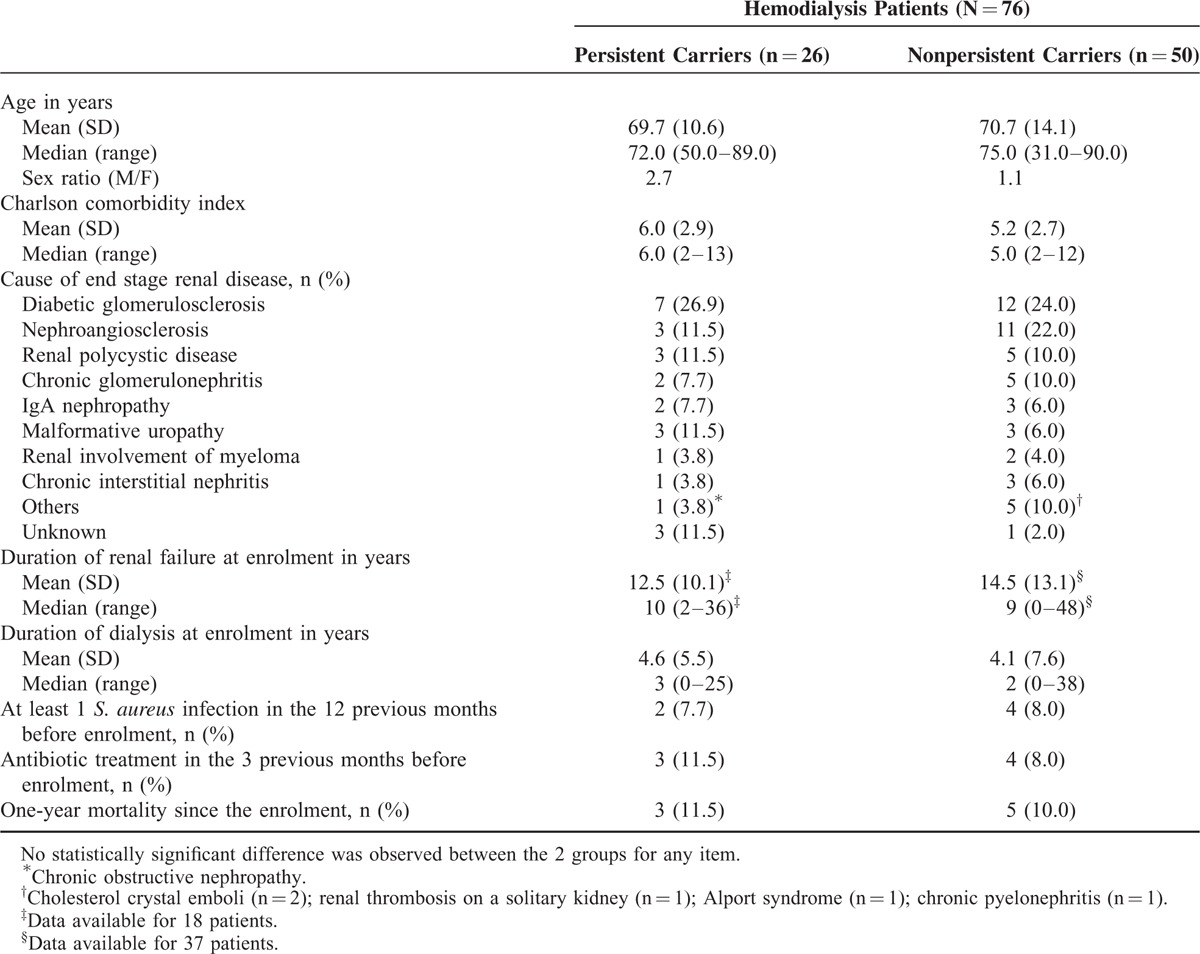
Characteristics of the Hemodialysis Patients According to the *S. aureus* Nasal Carriage Status as Determined by the Algorithm Based on 1 or 2 Nasal Samples

### Risk of *Staphylococcus aureus* Infection in HPs

The 76 HPs were followed up during a whole year after the last sampling episode, except for 1 patient who died after 11 months of follow-up. Eighteen *S. aureus* infections were recorded; Table 4 displays their characteristics and treatment in persistent and nonpersistent carriers according to the algorithm based on 1 or 2 nasal samples. No statistically significant difference was observed between the 2 groups for any of the listed items.

**TABLE 4:**
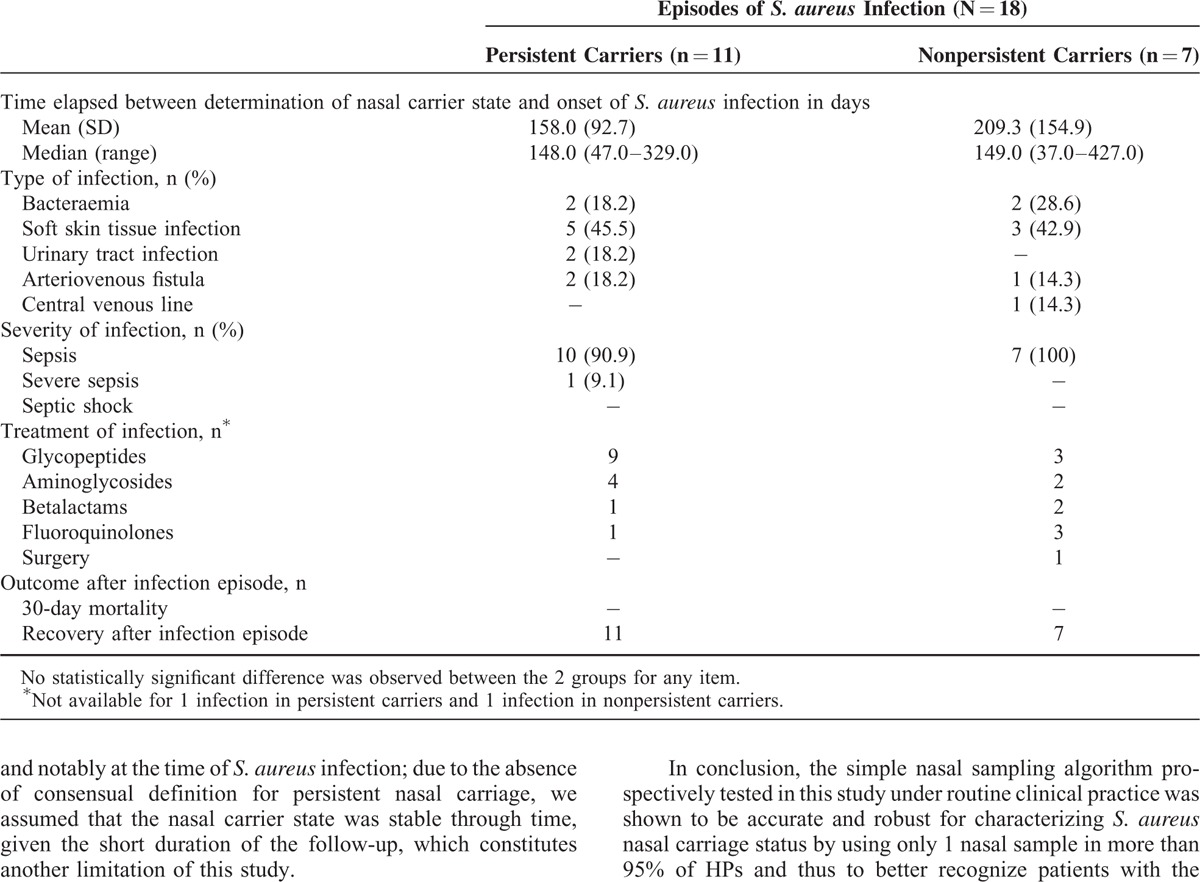
Characteristics of *S. aureus* Infections in Hemodialysis Patients According to the *S. aureus* Nasal Carriage Status as Determined by the Algorithm Based on 1 or 2 Nasal Samples

As shown in Table 5, the algorithm based on culture or qPCR was found able to characterize reliably the persistent nasal carriers at higher risk of *S. aureus* infection. By using both strategies, persistent carriers exhibited a statistically significant higher risk of *S. aureus* infection than nonpersistent ones (Table 5).

**TABLE 5:**
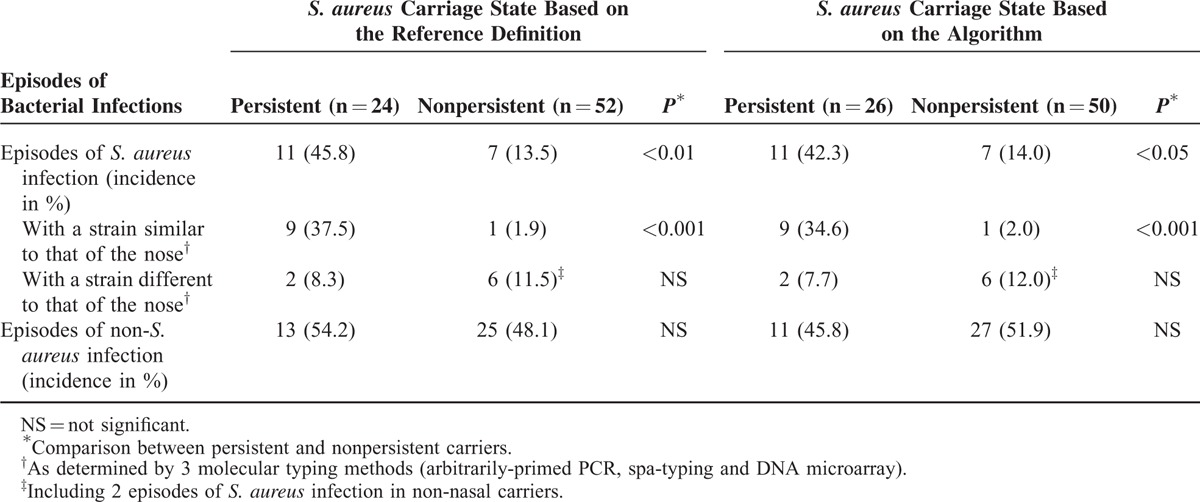
Bacterial Infections in Hemodialysis Patients According to the Nasal Carriage Status Determined by the Reference Definition or by the Algorithm Based on 1 or 2 Nasal Samples

Three typing methods were used for analyzing endogenous versus exogenous *S. aureus* infections. By using both strategies, persistent nasal carriers of *S. aureus* were found to have a statistically significant higher risk of endogenous infection than nonpersistent nasal carriers (Table 5). In contrast, the number of bacterial infections caused by species other than *S. aureus* did not differ significantly between persistent and nonpersistent nasal carriers of *S. aureus* (Table 5).

## DISCUSSION

The *S. aureus*-persistent nasal carriers are considered at higher risk of *S. aureus* infection,^1^ but the nasal carrier status of patients is very difficult to determine in routine clinical practice. Van Belkum et al^11^ proposed a reclassification of nasal carriage by recognizing only the persistent and the nonpersistent nasal carriage of *S. aureus*. This classification is notably argued by the fact that the probability to detect an intermittent carrier increases with the number of nasal samples taken in the noncarrier group.^1^

To our knowledge, before our works, only 1 published study^9^ had described a reliable method, called the “culture rule,” for characterizing the *S. aureus* nasal carriage state by using qualitative culture on 2 nasal samples taken 7 days apart. The present study reports the prospective evaluation of a faster method based on a simple algorithm that was shown able to identify the nasal carriage state of *S. aureus* in routine clinical practice. This algorithm was described on retrospective specimens from healthcare workers^10^; it is based on the determination of the *S. aureus* load in 1 or, exceptionally, 2 nasal specimens. In the present study, the nasal samplings were performed by a large number of healthcare workers including nurses, medical doctors, and students just orally informed of the sampling procedure. Despite this heterogeneity, the algorithm was shown to be accurate for distinguishing, in routine clinical practice, persistent from nonpersistent carriers of *S. aureus* by using only 1 nasal sample in most of the cases. The performances of the algorithm were found to be close to those of the reference method based on at least 7 sampling episodes. A small proportion of samples were misclassified as persistent with the algorithm, whereas they corresponded to intermittent carriers with the reference method, which could lead to overestimate the number of persistent carriers; in contrast, the opposite situation, which could result in neglecting true persistent carriers, was exceptional (Figure 1).

Of interest, a fully automated qPCR method could be used for quantifying the nasal load of *S. aureus* with a result available in less than 2 hours and performances very similar to those of quantitative culture. It is important to notice that the sampling step has an impact on the quantification of the *S. aureus* load; we both recommend the use of nylon flocked swabs that increase the recovered bacterial load^16^ and swab wetting with normal saline solution before nasal sampling.^19^

This algorithm exhibits several advantages: the *S. aureus* nasal carrier state can be determined very shortly (less than 2 hours by molecular biology and less than 24 hours by bacterial culture), with only 1 nasal sample in more than 95 % of cases; the performances of the algorithm in terms of sensitivity and specificity are similar to that of the reference method; the cost of this determination is significantly cheaper and less invasive as compared with the reference method^10^; and finally, the sampling can be performed easily by a variety of nursing personnel without impacting the quality of the results.

Two previous studies performed in patients undergoing peritoneal dialysis^7^ and in orthopedic surgery^8^ demonstrated that persistent nasal carriers of *S. aureus* have an increased risk of *S. aureus* infection by comparison with intermittent carriers and noncarriers. The results of the present study show for the first time that *S. aureus*-persistent nasal carriage increases significantly the risk of staphylococcal infection in HPs, whereas no difference was observed for infection with other bacterial species. Interestingly, the persistent nasal carriers were shown by using molecular typing methods to exhibit a statistically significant higher risk to be infected by the *S. aureus* strain recovered from their nose than nonpersistent ones.

Decolonization using mupirocin has been proven effective for reducing *S. aureus* infection rate in both HPs and chronic peritoneal dialysis patients.^2^ However, the modality of administration of this drug remains controversial^20^ because of the risk of emergence of antimicrobial resistance in *S. aureus* isolates.^3^ By using the proposed algorithm on a routine basis for the characterization of nasal carriage state in HPs, decolonization strategies could be limited to persistent nasal carriers who exhibit the highest risk of staphylococcal infection with their nasal strain, which implies an easy and early targeting of at-risk patients and would result in an important reduction of mupirocin consumption. In the present study, *S. aureus* nasal carriage was detected in approximately two-third of the patients. According to the rule defined just above, a decolonization protocol would have been applied to only half of them, those found to be persistent carriers, thus at higher risk of *S. aureus* infection with their own strain, but would have been avoided in nonpersistent carriers with a lower risk of *S. aureus* infection and mostly due to exogenous strains.

The main limitation of the study lies in the absence of verification of the nasal carrier state during the 1-year follow-up and notably at the time of *S. aureus* infection; due to the absence of consensual definition for persistent nasal carriage, we assumed that the nasal carrier state was stable through time, given the short duration of the follow-up, which constitutes another limitation of this study.

In conclusion, the simple nasal sampling algorithm prospectively tested in this study under routine clinical practice was shown to be accurate and robust for characterizing *S. aureus* nasal carriage status by using only 1 nasal sample in more than 95% of HPs and thus to better recognize patients with the highest risk of *S. aureus* infection. This approach could be considered as a simpler alternative to the “culture rule” for determining the carriage status of HPs on a routine basis. There is still a need for further clinical studies aimed at determining whether the same strategy could be useful to prevent infections in other clinical conditions than hemodialysis, including orthopedic surgery.
